# Reciprocal Alterations in Osteoprogenitor and Immune Cell Populations in Rheumatoid Synovia

**DOI:** 10.3390/ijms232012379

**Published:** 2022-10-16

**Authors:** Katarina Barbarić Starčević, Nina Lukač, Mislav Jelić, Alan Šućur, Danka Grčević, Nataša Kovačić

**Affiliations:** 1 Department of Orthopaedic Surgery, University Hospital Centre Zagreb, 10000 Zagreb, Croatia; 2 Laboratory for Molecular Immunology, Croatian Institute for Brain Research, University of Zagreb, School of Medicine, 10000 Zagreb, Croatia; 3 Department of Anatomy, University of Zagreb, School of Medicine, 10000 Zagreb, Croatia; 4 Department of Physiology and Immunology, University of Zagreb, School of Medicine, 10000 Zagreb, Croatia

**Keywords:** rheumatoid arthritis, osteoprogenitors, flow cytometry

## Abstract

Rheumatoid arthritis (RA) is chronic, autoimmune joint inflammation characterized by irreversible joint destruction. Besides increased resorption, destruction is a result of decreased bone formation, due to suppressed differentiation and function of the mesenchymal lineage-derived osteoblasts in inflammatory milieu. In this study, we analyzed the cellular composition of synovial tissue from 11 RA and 10 control patients harvested during planned surgeries in order to characterize resident synovial progenitor populations. Synovial cells were released by collagenase, and labeled for flow cytometry by two antibody panels: 1. CD3-FITC, CD14-PE, 7-AAD, CD11b-PECy7, CD235a-APC, CD19-APCeF780; and 2. 7-AAD, CD105-PECy7, CD45/CD31/CD235a-APC, and CD200-APCeF780. The proportions of lymphocytes (CD3^+^, CD19^+^) and myeloid (CD11b^+^, CD14^+^) cells were higher in synovial tissue from the patients with RA than in the controls. Among non-hematopoietic (CD45^−^CD31^−^CD235a^−^) cells, there was a decrease in the proportion of CD200^+^CD105^−^ and increase in the proportion of CD200^−^CD105^+^ cells in synovial tissue from the patients with RA in comparison to the control patients. The proportions of both populations were associated with inflammatory activity and could discriminate between the RA and the controls.

## 1. Introduction

Rheumatoid arthritis (RA) is chronic, autoimmune joint inflammation, characterized by synovial thickening, due to the proliferation of fibroblast-like synoviocytes (FLS), synovial infiltration by inflammatory cells, and the subsequent increased production of pro-inflammatory cytokines [1,2,3,4]. Persistent inflammation leads to joint destruction and results in permanent disability. Destruction of the underlying bone is mediated by enhanced homing and the activation of bone-resorbing osteoclasts [5,6,7], as well as impaired osteoblast differentiation and function [1,8,9]. The osteoblast differentiation and activity in RA are negatively affected by inflammatory milieu through the inhibition of Runx2 expression [10,11] or the induction of antagonists of osteoblast differentiation, such as DKK1 or BMP3 [12,13].

Mesenchymal lineage progenitor cells, with the ability to differentiate into bone and cartilage, are, among other sites, also present in synovia, and can be identified by a number of surface markers, such as transferrin receptor (CD73), endoglin (CD105), CD90, CD51, CD200, podoplanin (PDPN), stem cell antigen-1 (SCA-1), endolyn (CD164), low-affinity nerve growth factor receptors (CD271), platelet-derived growth factor receptors (PDGFR, CD140), and stromal cell surface marker-1 (STRO-1) [14]. Many of these markers have been characterized in cells expanded in vitro, which is associated with their upregulation, due to their role in cell adhesion. Therefore, the identification of a certain marker in a population of cells expanded in vitro does not necessarily reflect their expression in resident cells in vivo. Using a modified panel proposed by Chan and co-workers [15], we have previously described the loss of non-hematopoietic cells expressing CD200 in the synovial compartments of mice with antigen-induced arthritis (AIA) [16]. According to Chan and co-workers, murine skeletal non-hematopoietic (CD45^−^TER119^−^CD31^−^) progenitors expressing CD51 and CD200 corresponded to the earliest skeletal progenitors, so their reduction in arthritis might have reflected impaired osteogenic and chondrogenic regeneration [16]. In addition, the proportions of CD200^−^ cells expressing CD105, which, according to Chan and co-workers, represented committed bone and cartilage progenitors, were not affected in murine arthritis. The aim of the present study was to identify whether these alterations documented in murine arthritis were paralleled by alterations in synovial tissue from patients with severe forms of RA that underwent surgical treatment. We assessed the frequencies of cells belonging to main hematopoietic lineages, as well as proportions of resident synovial cells expressing CD200, CD105, or both markers in RA patients, and compared them to synovial cells from control patients that underwent surgery, due to non-inflammatory joint derangement, and assessed the association of their frequencies with inflammatory activity.

## 2. Results

**Figure 1:**
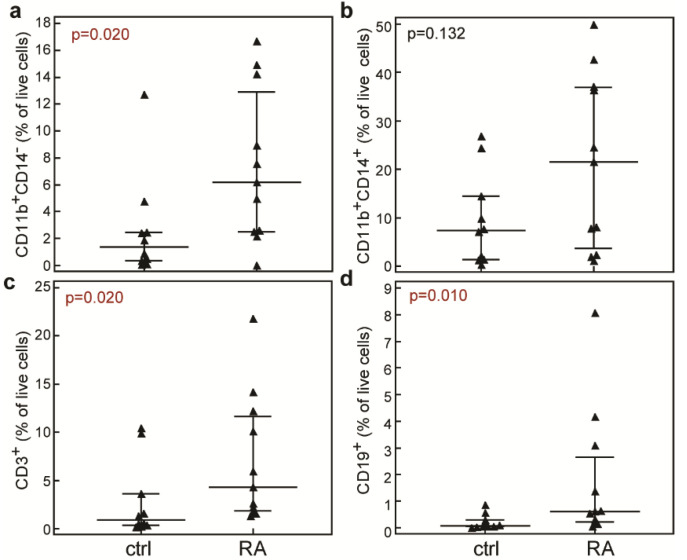
Proportions of main hematopoietic lineages in synovial tissue from control patients (ctrl) and patients with rheumatoid arthritis (RA). Single cell suspensions were stained with anti-human CD3-FITC, CD11b-PE, CD14-PECy7, CD235a-APC, and CD19-APCeF780 antibodies. Dead cells were excluded by binding of 7-AAD. Proportions of CD11b^+^CD14^−^ myeloid cells (**a**), CD11b^+^CD14^+^ myeloid cells (**b**), T-lymphocytes (CD3^+^, (**c**)) and B-lymphocytes (CD19^+^, （**d**)) were determined amongst single live cells and are shown as individual patient values (markers). Horizontal lines and bars are median and IQR; statistical significance is shown on plots (*p* < 0.05, Mann–Whitney test).

We first analyzed the distribution of the main hematopoietic populations in synovial tissue. Hematopoietic cell populations were delineated amongst single, live cells (7-AAD^−^) according to the gating strategy presented in Figure S1. Myeloid lineage was delineated according to the expression of CD11b and CD14, while T-lymphocytes (CD3^+^) and B-lymphocytes (CD19^+^) were delineated according to staining for the respective surface markers and scatter properties. As expected, we determined higher proportions of the main hematopoietic populations in synovial tissue from the patients with RA in comparison to the patients undergoing arthroscopy due to internal derangement of the knee (Figure 1). The proportion of CD11b^+^CD14^−^ cells was significantly higher in synovial tissue from the RA patients (6.16 [2.51–12.89]%) in comparison to the control patients (1.36 [0.36–2.47]%, *p* = 0.020, Mann-Whitney test, Figure 1a). Although the median proportion of CD11b^+^CD14^+^ myeloid cells was higher in RA patients in comparison to controls, the difference was not significant (21.50 [3.70–36.93]% in RA group vs. (7.37 [1.35–14.40]% in control group, *p* = 0.132, Mann–Whitney test, Figure 1b). The synovial tissue from patients with RA also contained significantly higher proportions of T-lymphocytes (CD3^+^, 4.32 [1.87–11.68]%, Figure 1c) and B-lymphocytes (CD19^+^, 0.61 [0.21–2.65]%, Figure 1d) than the synovial tissue from control patients (CD3^+^, 0.92 [0.34–3.63]%, *p* = 0.020; CD19^+^, 0.08 [0.04–0.3]%, *p* = 0.010, Mann–Whitney test).

**Figure 2:**
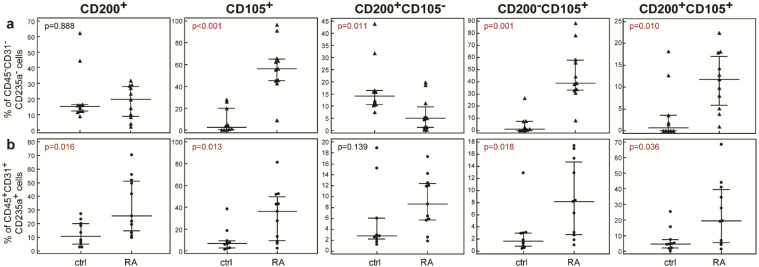
Proportions of non-hematopoietic (**a**) and hematopoietic (**b**) cells in synovial tissue from controls (ctrl) and patients with rheumatoid arthritis (RA), expressing markers CD200 and CD105. Single cell suspensions were stained with anti-human CD105-PECy7, CD45-APC, CD235a-APC, CD31-APC and CD200-biotin, and streptavidin-APCeF780 antibodies. Dead cells were excluded by binding of 7-AAD. Proportions of CD200^+^, CD105^+^, CD200^+^CD105^−^, CD200^−^CD105^+^, and CD200^+^CD105^+^ cells were determined amongst non-hematopoietic CD45^−^CD31^−^CD235a^−^ (**a**) and hematopoietic CD45^+^CD31^+^CD235a^+^ cells (**b**). Proportions are shown as individual values (markers), horizontal lines and bars are median and IQR; statistical significance is shown on plots (*p* < 0.05, Mann–Whitney test).

The staining with the first antibody (leukocyte) panel confirmed increased inflammatory infiltration of the rheumatoid synovia, so we further aimed to assess the proportion of cells expressing potential skeletal progenitor markers CD200 and CD105 with the second (mesenchymal lineage) antibody panel. The expression of both markers is not specific for non-hematopoietic populations, and there are reports of their expression in hematopoietic cell subsets [17,18,19]. Therefore, we simultaneously assessed their expression among non-hematopoietic (CD45^−^CD31^−^CD235a^−^) and hematopoietic (CD45^+^CD31^+^CD235a^+^) cells delineated amongst single, live (7-AAD^−^) cells, according to the gating strategy presented in Figure S1. In both populations, we established significantly different expression patterns in synovial tissue from patients with RA in comparison to patients undergoing arthroscopy due to joint derangement (Figure 2). The total proportion of CD45^−^CD31^−^CD235a^−^CD200^+^ cells was similar in the RA patients (19.60 [8.70–28.02]%) and controls (15.04 [12.17–16.40]%, *p* = 0.888, Mann–Whitney test, Figure 2a), and the total proportion of CD45^−^CD31^−^CD235a^−^CD105^+^ cells was higher in the synovial tissue from RA patients (56.20 [45.25–65.22]%) in comparison to the control group (2.43 [0.22–19.97]%, *p* < 0.001, Mann–Whitney test, Figure 2a). When non-hematopoietic populations were further subdivided according to the expression of one or both markers, it became apparent that the proportion of CD45^−^CD31^−^CD235a^−^CD200^+^CD105^−^ cells was clearly reduced in the synovia of RA patients in comparison to the controls (5.02 [1.28–9.65]% in RA vs. (14.05 [10.70–16.40]% in controls, *p* = 0.011, Mann–Whitney test, Figure 2a). On the other hand, the CD45^−^CD31^−^CD235a^−^ CD200^−^CD105^+^ cells were highly enriched in the synovia of patients with RA in comparison to the controls (39.10 [33.15–57.80]% in RA vs. (1.07 [0.00–7.27]% in control group, *p* = 0.001, Mann–Whitney test, Figure 2a). A similar trend was observed for the CD45^−^CD31^−^CD235a^−^ CD200^+^CD105^+^ cells (11.80 [5.84–17.00]% in RA vs. 0.71 [0.00–3.57]% in controls, *p* = 0.010, Mann–Whitney test, Figure 2a). 

Conversely, among the hematopoietic lineages, the proportions of CD45^+^CD31^+^CD235a^+^CD105^+^ cells and CD45^+^CD31^+^CD235a^+^CD200^+^ cells were both increased in the synovial tissue from RA patients in comparison to the control group (Figure 2b). Both CD105^+^ subpopulations (CD200^−^CD105^+^ and CD200^+^ CD105^+^) were significantly higher in the RA samples, although the differences were less pronounced in the non-hematopoietic cells, while only CD200^+^CD105^+^ hematopoietic cells were more abundant in the RA samples, and the proportion of CD200^+^CD105^−^ cells was not different from the controls (Figure 2b).

After we established differences in the proportions of cells expressing CD105, CD200 or both markers in the synovial tissue, we aimed to assess their potential association with the intensity of synovial infiltration by inflammatory cells and systemic markers of inflammatory activity, such as the erythrocyte sedimentation rate or concentration of C-reactive protein. The proportion of non-hematopoietic CD105^+^ and CD200^−^CD105^+^ cells was clearly positively associated with the serum concentration of C-reactive protein, as well as with the proportion of infiltrating synovial T- (CD3^+^) and B-lymphocytes (CD19^+^) (Table 1, Spearman’s rank correlation). Conversely, the non-hematopoietic CD200^+^CD105^−^ population was clearly negatively associated with the concentration of C-reactive protein (Table 1, Spearman’s rank correlation), and not with synovial inflammatory infiltration. The proportions of hematopoietic CD200^+^ and CD105^+^ cells were all positively associated with the lymphocytic or myeloid cell (CD11b^+^CD14^−^, CD11b^+^CD14^+^) infiltration of synovial tissue, but not associated with the markers of systemic inflammatory activity (Table 1, Spearman’s rank correlation). 

**Figure 3:**
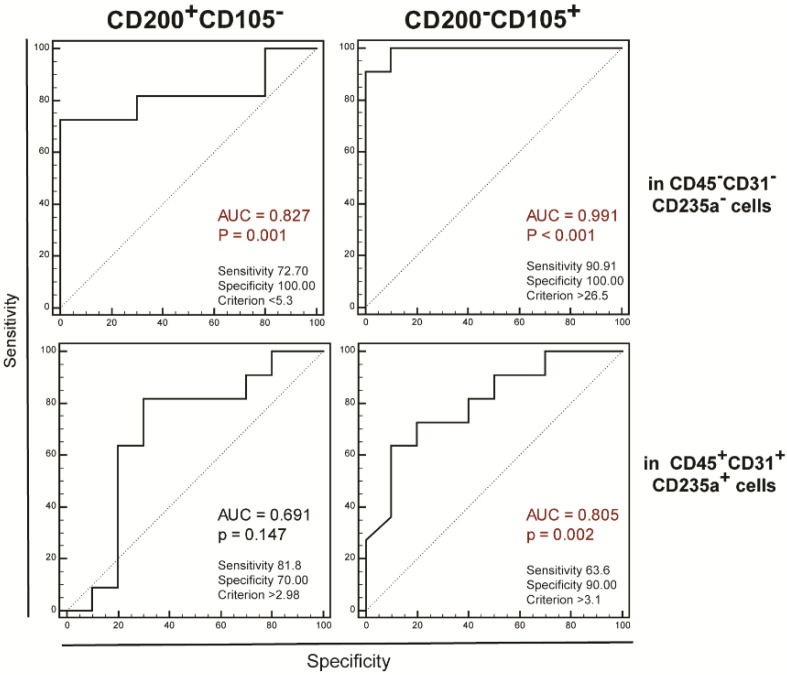
Ability of proportions of CD200^+^CD105^−^ and CD200^−^CD105^+^ populations to discriminate between RA patients and controls was assessed by receiver operating characteristic (ROC) curves showing discriminatory ability of proportions of CD200^+^CD105^−^ and CD200^−^CD105^+^ amongst non-hematopoietic (**upper panels**) and hematopoietic (**lower panels**) cells between arthritic and control samples. Sensitivity, specificity, criterion point, *p* value, and area under curve (AUC) are shown on plots.

To assess the ability of the proportions of both populations to discriminate the patients with RA from the control patients without joint inflammation, we performed ROC curve analysis and found that the control and patient groups could be best distinguished based on the proportions of synovial non-hematopoietic CD200^+^CD105^−^cells (AUC = 0.827, *p* = 0.001) and non-hematopoietic CD200^−^CD105^+^ cells (AUC = 0.991, *p* < 0.001, Figure 3).

## 3. Discussion

This study provided pilot data demonstrating the altered proportions of resident synovial non-hematopoietic subpopulations, classified according to the expression of two potential osteoprogenitor markers, CD200 and CD105, and their association with RA, inflammatory activity (CRP), and the degree of synovial inflammatory infiltration.

The proportion of the non-hematopoietic (CD45^−^CD31^−^CD235a^−^) CD200^+^CD105^−^ population was reduced in the synovial tissue of patients with RA. Reduction in these cells is associated with the diagnosis of RA and with increased systemic inflammatory activity. CD200 is a type I transmembrane glycoprotein from the immunoglobulin family, whose expression has been documented in a variety of cells [18,20,21]. It has the ability to suppress the immune response in various conditions, including arthritis and autoimmune diseases [21,22], and participates in osteoclast fusion and differentiation [17]. The expression of CD200 in human bone marrow-derived mesenchymal stem cells is related to their ability to suppress TNF-α secretion from macrophages [23]. As a skeletal progenitor marker, CD200 has so far been characterized by Chan and co-workers in murine bones [15]. In addition to non-hematopoietic cells, the hematopoietic cells of myeloid lineage, as well as lymphocytes, express CD200 [18,20,21]. Among studies of human samples with RA, Ren and co-workers described decreased proportions of CD200^+^ peripheral blood mononuclear cells in comparison to the controls, which were restored by treatment with infliximab and methotrexate [18]. The same study reported an increased number of cells that were immunohistochemically positive for CD200 in the synovia from RA patients in comparison to the control synovial samples. Our results also showed an increase in CD200^+^ expression amongst hematopoietic cells, which is in line with this report. The next finding in our study was an increase in the proportion of non-hematopoietic cells expressing endoglin (CD105) in rheumatoid synovia, which was associated with the diagnosis of RA and with increased systemic inflammatory activity. Endoglin is a co-receptor for ligands of the transforming growth factor (TGF)-β family, and is known for its role in the regulation of endothelial cell proliferation and function. It is also expressed in many other cell types, yet its precise role in their development and function still needs to be addressed [24]. It represents one of the common markers used for the characterization and isolation of mesenchymal cells [25,26]. Moreover, CD105 also seem to be involved in fibroblast activation and proliferation, as well as increased TGF-β signaling [24]. Since fibroblasts and mesenchymal lineage cells share many phenotypic and morphological characteristics, it is not possible to delineate these cell types solely on the expression of CD105. In addition, CD105 affects the differentiation and immunoregulatory properties of murine mesenchymal stem cells, while in human cells, the lack of CD105 expression seems to enhance their immunoregulatory capabilities [27,28]. In human rheumatoid synovium, CD105 has been immunohistochemically detected in pericytes, vascular smooth muscle cells, intimal cells, subintimal macrophages and fibroblasts [19], and in murine bones CD105 delineates committed bone and cartilage progenitors [15]. According to our transcriptome comparation of the sorted murine non-hematopoietic CD200^+^CD105^−^ and CD200^−^CD105^+^ synovial populations from the AIA model, BMP, Wnt and TGF-β pathways were only overexpressed in the CD200^+^CD105^−^, and were underexpressed in the CD200^−^CD105^+^, so CD200^−^CD105^+^ cells could potentially represent hypertrophic synoviocytes in murine and human RA [29].

In this study, we attempted to assess resident synovial cell phenotypes directly, without the prior expansion of cells in vitro, where interaction with plastic surfaces can alter their surface marker expression, and often results in the increased expression of many mesenchymal markers. However, such an approach imposed some significant limitations to our study. First, the number of RA patients seeking surgical treatment is constantly decreasing, due to earlier diagnosis and better therapeutic management, which limited patient recruitment to the study. Second, we assessed samples from patients with severe RA, because earlier stages typically do not require surgey, so we were not able to draw conclusions about earlier stages of the disease. Third, patients were receiving different therapeutics (Table 2), which could have potentially affected the results. Due to the limited number of participants and the diverse therapeutics used, the impact of therapeutics could not be assessed. We believe that, despite the limitations, our findings are of interest, but should be confirmed by studies with more subjects.

## 4. Materials and Methods

### 4.1. Patients

A total of 21 patients were included in the study (5 male and 16 female, Table 2). The patients were admitted to the Department of Orthopedic Surgery at the Zagreb University Hospital Center between May 2017 and May 2022 with the diagnosis of rheumatoid arthritis (RA, N = 11), or requiring arthroscopy of the knee, due to internal derangement (control group, N = 10). The patients with RA significantly differed from the controls in their systemic indicators of inflammation (erythrocyte sedimentation rate and C-reactive protein concentration, Table 2).

The patients were recruited after obtaining institutional ethics approval, number 380-59-10106-16-20/289, issued by the Ethics Committee of the University of Zagreb School of Medicine, and after signing the informed consent document. All procedures were carried out according to the Declaration of Helsinki. All experimental procedures were approved by the Ethics committee of the University of Zagreb School of Medicine. The samples were patches of synovial tissue removed during planned surgical procedures for patients with RA and post-traumatic joint lesions. All patients were operated under regional anesthesia. Patients with RA were treated by synovectomy of the affected joint. In the control group, patients were treated with meniscectomy, menisceal suture, reconstruction of the anterior cruciate ligament of the knee, excision of the parapatellar fold or ganglion cyst. During the surgical procedures, a piece of synovial tissue was harvested with a biopsy grasp, immediately placed in a cold (+4 °C) 0.1M phosphate buffered saline (PBS), and transported to the laboratory for processing within 1 h. The samples were kept on ice during transportation. Samples were harvested before the surgical procedures to prevent damage to the synovial tissue.

### 4.2. Flow Cytometry

The synovial tissue was cleaned from the adjacent bone and cartilage fragments, or fat tissue, placed in a Petri dish mixed with a 1.5 mg/mL solution of Collagenase type IV form Clostridium histolyticum (Sigma #C-5138, Sigma-Aldrich, St. Louis, MO, USA) in 0.1M of PBS, and incubated for 1 h at 37 °C. Cell suspension was prepared by suspending the contents of the Petri dish for a minimum of 5 times through a 2 mL syringe and 23G needle, and then transferred to FACS tubes through a 100 μm cell strainer. The cells were washed with 0.1M of PBS to completely remove collagenase. After blocking the non-specific antibody binding using anti-human CD16/CD32 (eBiosciences, San Diego, CA, USA, 1:100) for 5 min at RT, the cells were labeled with the following antibodies: anti-human CD3 FITC (eBiosciences, 1:100), anti-human CD14 PE (eBiosciences, 1:100), anti-human CD11bPECy7 (eBiosciences, 1:100), anti-human CD235aAPC (eBiosciences, 1:100), anti-human CD19 APCeF780 (BioLegend, 1:100), anti-human CD105-PE (eBiosciences, 1:200), anti-human CD31 APC (eBiosciences, 1:100), anti-human CD45APC (eBiosciences, 1:100), and anti-human CD200-biotin (BioLegend, San Diego, CA, USA, 1:100). Samples were further incubated with a Streptavidin APC-eFluor^®^ 780 (eBioscience, 1:400) for 25 min at 4°. Dead cells were excluded by the binding of 7-amino-actinomycin D (7-AAD, BioLegend). Scatter and fluorescent signals were acquired on an Attune (Thermo Fisher Scientific, Waltham, Massachusetts, USA) instrument, and analyzed by FlowJo software (FlowJo, v10, Ashland, OR, USA) according to the gating strategy presented in Figure S1. The hematopoietic cell populations were briefly delineated amongst single, live cells (7-AAD^−^). Myeloid lineage was delineated according to the expression of CD11b and CD14, whereas T- (CD3^+^) and B-lymphocytes (CD19^+^) were delineated according to staining for the respective surface markers and scatter properties. Non-hematopoietic (CD45^−^CD31^−^CD235a^−^) and hematopoietic (CD45^+^CD31^+^CD235a^+^) cells were further subdivided according to the expression of the markers CD200 and CD105. Positive populations were delineated according to the signals of non-stained cells (NS).

Statistical analysis was performed using MedCalc (version 20.006; MedCalc Software Ltd., Ostend, Belgium). Data were presented as median ± IQR, and the differences were assessed using the Mann–Whitney test. Association between the variables was assessed using Spearman’s rank correlation. The statistical significance was set to *p* < 0.05.

## 5. Conclusions

This study found a reduction in the proportion of CD200^+^CD105^−^ non-hematopoietic cells and an accumulation of the CD105^+^CD200^−^ non-hematopoietic cells in synovial tissue from patients with RA. The proportion of CD200^+^CD105^−^ was negatively associated with systemic inflammatory activity and synovial infiltration with inflammatory cells, and the proportion of CD105^+^CD200^−^ was positively associated with systemic inflammatory activity and synovial infiltration with inflammatory cells. Since our translational study was performed on a limited number of samples, the results should be confirmed by future phenotypic and functional studies.

## Figures and Tables

**Table 1:** Associations of proportions of non-hematopoietic and hematopoietic synovial cells classified according to their expression of CD200 and CD105, with frequencies of major hematopoietic lineages in the synovial tissue and diagnostic markers of inflammatory activity.

Parent Population	Subpopulation		CD19^+^ *	CD3^+^ *	CD11b^+^CD14^−^ *	CD11b^+^CD14^+^ *	CRP **	ESR ***
Non- hematopoietic cells (CD45^−^ CD31^−^ CD235a^−^)	Total CD200^+^	ρ *p*	0.251 0.2717	**0.433** **0.0498**	−0.036 0.8778	0.021 0.9287	−0.240 0.3895	−0.127 0.6278
	Total CD105^+^	ρ *p*	**0.624** **0.0025**	**0.515** **0.0169**	0.364 0.1044	0.279 0.2201	**0.558** **0.0306**	0.365 0.1503
	CD200^+^CD105^−^	ρ *p*	−0.157 0.4980	−0.050 0.8295	−0.375 0.0941	−0.272 0.2338	**−0.601** **0.0178**	−0.370 0.1432
	CD200^−^CD105^+^	ρ *p*	**0.568** **0.0072**	**0.454** **0.0388**	0.401 0.0720	0.265 0.2452	**0.687** **0.0047**	0.395 0.1168
	CD200^+^CD105^+^	ρ *p*	**0.515** **0.0169**	**0.667** **0.0010**	0.275 0.2285	0.242 0.2909	0.224 0.4218	0.237 0.3594
Hematopoietic cells (CD45^+^ CD31^+^ CD235a^+^)	Total CD200^+^	ρ *p*	**0.569** **0.0071**	**0.671** **0.0009**	**0.674** **0.0008**	**0.835** ** < 0.0001**	0.366 0.1792	0.047 0.8585
	Total CD105^+^	ρ *p*	**0.484** **0.0261**	**0.608** **0.0035**	**0.630** **0.0022**	**0.790** ** < 0.0001**	0.372 0.1724	0.103 0.6929
	CD200^+^CD105^−^	ρ *p*	**0.504** **0.0198**	**0.499** **0.0213**	0.305 0.1794	0.314 0.1661	0.138 0.6248	0.114 0.6633
	CD200^−^CD105^+^	ρ *p*	**0.446** **0.0426**	**0.494** **0.0229**	**0.673** **0.0008**	**0.595** **0.0044**	0.495 0.0606	0.193 0.4572
	CD200^+^CD105^+^	ρ *p*	0.406 0.0675	**0.539** **0.0117**	**0.571** **0.0068**	**0.734** **0.0002**	0.263 0.3441	0.028 0.9141

ESR, erythrocyte sedimentation rate (mm/h); CRP, C-reactive protein (mg/L); ρ, Spearman rank correlation coefficient; *p*, statistical significance; *, N = 21; **, N = 15; ***, N = 17. Significant associations are emphasized in red.

**Table 2:** Selected demographic and clinical characteristics of control subjects and patients with rheumatoid arthritis (RA).

Characteristic	Control	RA
Number of subjects	10	11
Male/female	5/5	0/11
Age, years	56 [53–58]	57 [46.25–70.5]
Diagnosis (ICD-10)	M05 (N = 7), M06 (N = 3), M08 (N = 1)	M23 (N = 10)
ESR	6 [5–7.75], (N = 7)	18 [12–42] (N = 10) *
CRP	1.40 [0.85–3.63] (N = 7)	6.85 [4.20–41.65] (N = 8) *
Affected joints	Knee (N = 10)	Hand and wrist (N = 6), elbow (N = 1), knee (N = 3), hip (N = 1)
NSAIDs	n/a	6/11
DMARDs	n/a	6/11
Corticosteroids	n/a	9/11
Biological therapy	n/a	3/11

ESR, erythrocyte sedimentation rate (mm/h); CRP, C-reactive protein (mg/L); NSAIDs, non-steroid anti-inflammatory drugs; DMARDs, disease-modifying antirheumatic drugs; n/a, not applicable; ICD-10, International Classification of Diseases 10th revision; M05, seropositive rheumatoid arthritis; M06, other rheumatoid arthritis; M08, juvenile arthritis; M23, internal derangement of knee (diagnoses: parapatellar fold, ruptured meniscus, ganglionic cyst, cruciate ligament injuries). Values are presented as medians [interquartile range]. * Statistically significant difference from the control group, *p* < 0.05, Mann–Whitney test.

## Data Availability

Data are contained within the article or Supplementary Material. Any additional data are available from the corresponding author upon reasonable request.

## Supplementary Materials

The supporting information can be downloaded at: https://www.mdpi.com/article/10.3390/ijms232012379/s1.
