# Supplementary material for: Reciprocal Alterations in Osteoprogenitor and Immune Cell Populations in Rheumatoid Synovia
Source: Int J Mol Sci. 2022 Oct 16;23(20):12379. doi: 10.3390/ijms232012379 (PMC9604389; doi:10.3390/ijms232012379)
Supplement: Supplementary file 1 [file ijms-23-12379-s001.zip › ijms-1928257-supplementary.pdf]

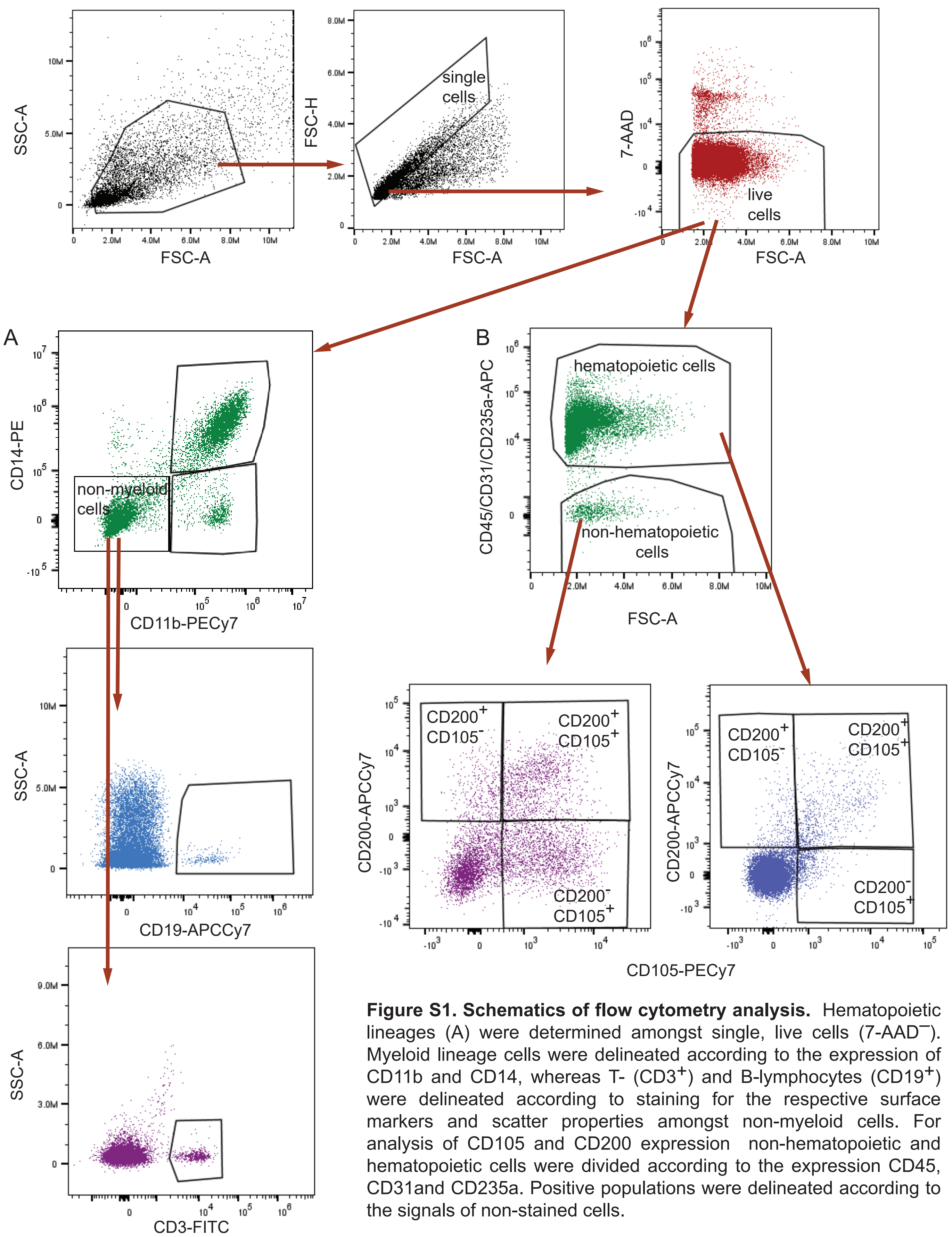

Figure S1. Schematics of flow cytometry analysis. Hematopoietic lineages (A) were determined amongst single, live cells (7-AAD^{-}). Myeloid lineage cells were delineated according to the expression of CD11b and CD14, whereas T- (CD3^{+}) and B-lymphocytes (CD19^{+}) were delineated according to staining for the respective surface markers and scatter properties amongst non-myeloid cells. For analysis of CD105 and CD200 expression non-hematopoietic and hematopoietic cells were divided according to the expression CD45, CD31and CD235a. Positive populations were delineated according to the signals of non-stained cells.

Lorem ipsum
